# Impact of Age on Endothelial Function of Saphenous Vein Grafts in Coronary Artery Bypass Grafting

**DOI:** 10.3390/jcm12175454

**Published:** 2023-08-22

**Authors:** Lars Saemann, Lena Wernstedt, Sabine Pohl, Markus Stiller, Jan Willsch, Britt Hofmann, Gábor Veres, Andreas Simm, Gábor Szabó

**Affiliations:** 1 Department of Cardiac Surgery, University Hospital Halle, Ernst-Grube-Straße 40, 06120 Halle (Saale), Germanygabor.veres@uk-halle.de (G.V.); andreas.simm@uk-halle.de (A.S.); gabor.szabo@uk-halle.de (G.S.); 2 Department of Cardiac Surgery, Heidelberg University Hospital, 69120 Heidelberg, Germany

**Keywords:** coronary artery bypass grafting (CABG), saphenous vein, aging, senescence, coronary artery disease, endothelium

## Abstract

Background: An intact and functionally preserved endothelial layer in the graft is crucial for myocardial perfusion and graft patency after coronary artery bypass grafting (CABG). We hypothesized that old age is a risk factor for decreased endothelial function of bypass grafts. Thus, we investigated the impact of age in patients treated with CABG on endothelial function in saphenous vein grafts. Methods: We mounted the saphenous vein graft segments of CABG patients < 70 (*n* = 33) and ≥70 (*n* = 40) years of age in organ bath chambers and exposed them to potassium chloride (KCl) and phenylephrine (PE) to test the receptor-independent and -dependent contractility, followed by exposure to acetylcholine (ACh) and sodium nitroprusside (SNP) to test the endothelial-dependent and -independent relaxation. Results: The maximal contraction induced by KCl (2.3 ± 1.8 vs. 1.8 ± 2 g) was stronger in patients ≥ 70 years of age. The relative contraction induced by PE in % of KCl (167 ± 64 vs. 163 ± 59%) was similar between groups. Patients aged < 70 years showed a higher endothelial-dependent relaxation induced by acetylcholine than patients ≥ 70 years (51 ± 27 vs. 42 ± 18%). The relaxation induced by SNP was similar between both groups. Conclusions: The endothelial function of saphenous vein bypass grafts decreases during aging. Thus, age should be considered when improving graft maintenance.

## 1. Introduction

Coronary artery bypass grafting (CABG) is the method of choice for the surgical treatment of coronary artery disease (CAD) [1]. Short-term as well as long-term results depend on graft viability and patency. An intact and functionally preserved endothelial layer in the graft is crucial for a high blood flow in the graft and, thus, for re-establishing myocardial perfusion and curing myocardial ischemia behind the stenosis. Additionally, endothelial dysfunction in saphenous vein grafts is associated with intimal thickening, which leads to long-term graft failure [2].

Patient age in cardiac surgery has increased over the past decades [3]. In Germany, the percentage of patients ≥ 80 years rose from 13.8% in 2012 to 20.7% in 2021 [3]. Furthermore, complications, morbidity, and mortality in CABG surgery combined with valve replacement surgery increase with age [4,5]. In a clinical restenosis risk model, age was also considered a relative risk factor for decreased graft patency after CABG [6]. Consequently, we hypothesized that old age is also a risk factor for the decreased endothelial function of bypass grafts. Saphenous vein grafts are the most frequently used conduits for CABG surgery [7]. Thus, we investigated the impact of age in patients treated with CABG on endothelial function in saphenous vein grafts.

## 2. Material and Methods

### 2.1. Patients

The present project is a prospective, observational trial. The study was approved by the institutional ethics committee of the medical faculty, Martin Luther University of Halle-Wittenberg, Germany. We consecutively included patients with saphenous vein harvesting for CABG and divided them into two groups: <70 and ≥70 years of age. Informed consent was obtained from all subjects involved in the study.

### 2.2. Harvesting and Collection of the Graft

According to our common institutional procedure, the saphenous vein was harvested endoscopically. Leftover segments of the harvested saphenous vein were immediately transferred into our institutional research facility, surrounded by the solution in which they were stored in the operating room. The storage solution was documented and chosen based on the surgeons’ preference. The patient data were collected prospectively from our hospital information system.

### 2.3. Preparation of the Graft

The harvested graft segment, collected from the operating room, was immediately placed in cold carbonized Krebs–Henseleit solution (KHS). We carefully removed the periadventitial fat and connective tissue under microscopic vision. Afterward, we cut the vessel segment into rings of 4 mm in length.

### 2.4. Functional Assessment

**Figure 1:**
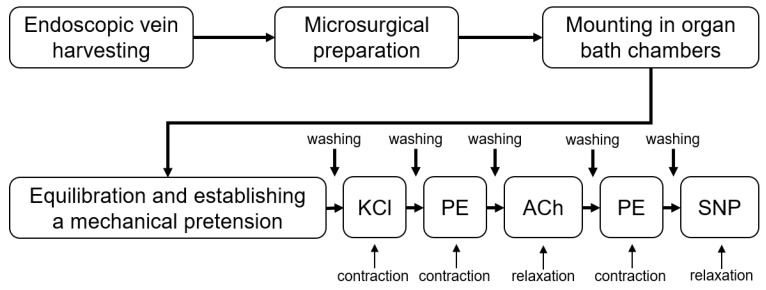
Functional assessment of saphenous vein grafts. ACh: Acetylcholine. KCL: Potassium chloride. PE: Phenylephrine. SNP: Sodium-nitroprusside.

Each vessel ring was mounted in a separate organ bath chamber (EMKA 4 Bath, EMKA Technologies S.A.S, Paris, France) filled with continuously carbonized normothermic (37 °C) KHS. Initially, the vessel rings were equilibrated for 20 min at 0.2 g (Figure 1). Then, the tension of the vessel was adjusted in steps of 0.75 g to a final pre-tension of 2.5 g over a time of 70 min, including repeated washing steps with fresh KHS to remove potential metabolites. To evaluate the vasomotor function, we first exposed the vessel rings to 80 mM potassium chloride (KCl) to test the maximal receptor-independent contractility. When a stable plateau was reached, we washed the chambers with fresh KHS and readjusted the tension to 2.5 g. With a gradually increasing concentration (10^−9^ to 10^−5^ M) of the α-adrenergic receptor agonist phenylephrine (PE), we induced vasoconstriction, followed by gradually increasing concentrations (10^−9^ to 10^−4^ M) of acetylcholine (ACh) to test the endothelial-dependent vasorelaxation. The ACh-induced vasorelaxation is the primary goal parameter of the study. If one ring did not reach at least 3.0 g of contraction after PE administration, this ring was excluded from the analysis because low PE-mediated contractions result in false-high ACh-mediated relaxations. If more than one ring did not reach 3.0 g of PE-mediated contraction, the patient was excluded from the study. For PE as well as for ACh, we calculated the pD_2_ value, which is the logarithmized value of the concentration which is needed to reach the half-maximal effect.

To investigate the endothelial-independent relaxation, we induced one further contraction by a single dose of PE (10^−7^), followed by gradually increasing sodium nitroprusside (SNP) concentrations (10^−10^ to 10^−4^ M). 

### 2.5. Statistics

IBM SPSS Statistics for Windows (version 20.0, IBM Corp., Armonk, NY, USA) was used to perform the statistical analyses. Data were presented as mean ± standard error (SEM). We used Graph Pad Prism (Version 9, Graph Pad Software Inc., San Diego, CA, USA) to perform a nonlinear curve fit. Data were compared using a χ^2^ test for categorical data or a two-tailed unpaired classical *t*-test in variance homogeneity and a Welch *t*-test in variance inhomogeneity. A *p* < 0.05 was considered statistically significant, and a *p* < 0.001 was considered highly significant.

## 3. Results

### 3.1. Patient Data Stratified by Age

We included *n* = 40 patients ≥70 years of age and *n* = 30 patients < 70 years of age (Table 1), with a comparable distribution of both sexes. The mean age in the group < 70 years was 62.4 ± 1.2 years, and the mean age in the group ≥ 70 years was 76.5 ± 0.6 years. Bodyweight (BW) was significantly different between patients ≥ 70 years (79.9 ± 2.3 kg) and <70 years (91.3 ± 3.0 kg). However, the body mass index was comparable between both groups. Different storage solutions, such as Tiprotec^®^ (Köhler Chemie, Bensheim, Germany), Ringer solution, or 0.9% NaCl solution, were used to store the grafts between harvesting and anatomization. Storage solutions, as well as graft diameters, were comparable between both groups. Comorbidities, such as arterial hypertension (52.1 vs. 42.5%), NYHA classification, LVEF, and prevalence of PAD (2.7 vs. 5.5%), did not differ significantly between both groups. Metabolism-associated disorders, such as obesity, diabetes mellitus, and hyperlipoproteinemia (26 vs. 17.8%), did not differ significantly. The prevalence of chronic kidney insufficiency was also comparable between both groups. Patient characteristics regarding administering oral antidiabetics, antiplatelet drugs, novel oral anticoagulation drugs, diuretics, and statins did not show major differences.

### 3.2. Vasomotor Function Stratified by Age

**Figure 2:**
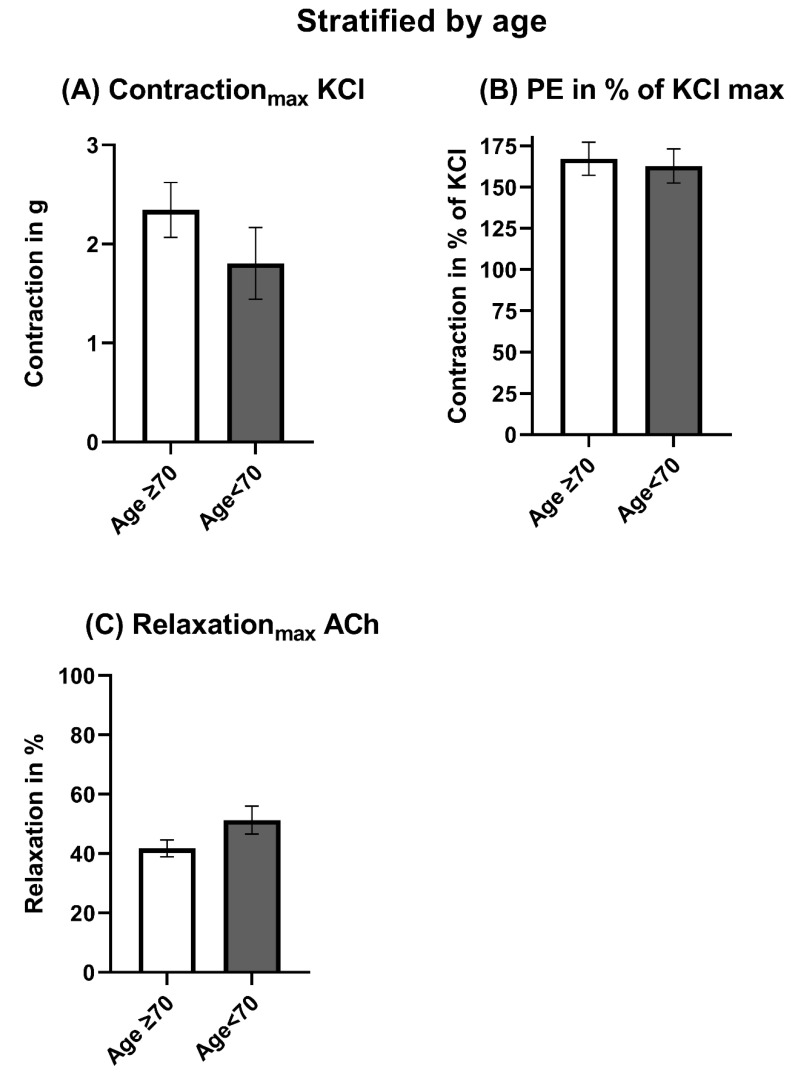
Maximal endothelial-dependent vasomotor response stratified by age. ACh: Acetylcholine. KCl: Potassium chloride. PE: Phenylephrine. The data were compared using a two-tailed unpaired classical *t*-test in case of variance homogeneity and a Welch *t*-test in case of variance inhomogeneity.

**Figure 3:**
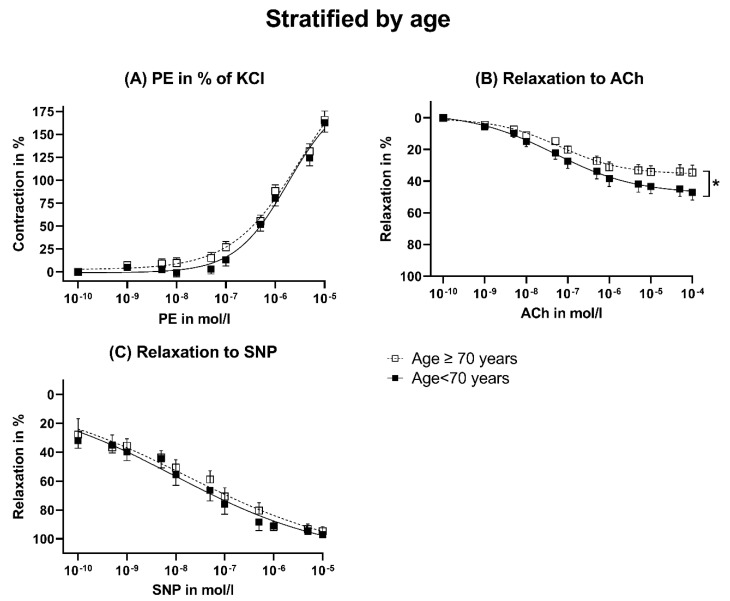
Dose–response curves stratified by age. ACh: Acetylcholine. KCl: Potassium chloride. PE: Phenylephrine. SNP: Sodium nitroprusside. The data were compared using a two-tailed unpaired classical *t*-test in case of variance homogeneity and a Welch *t*-test in case of variance inhomogeneity. * *p* < 0.05.

**Figure 4:**
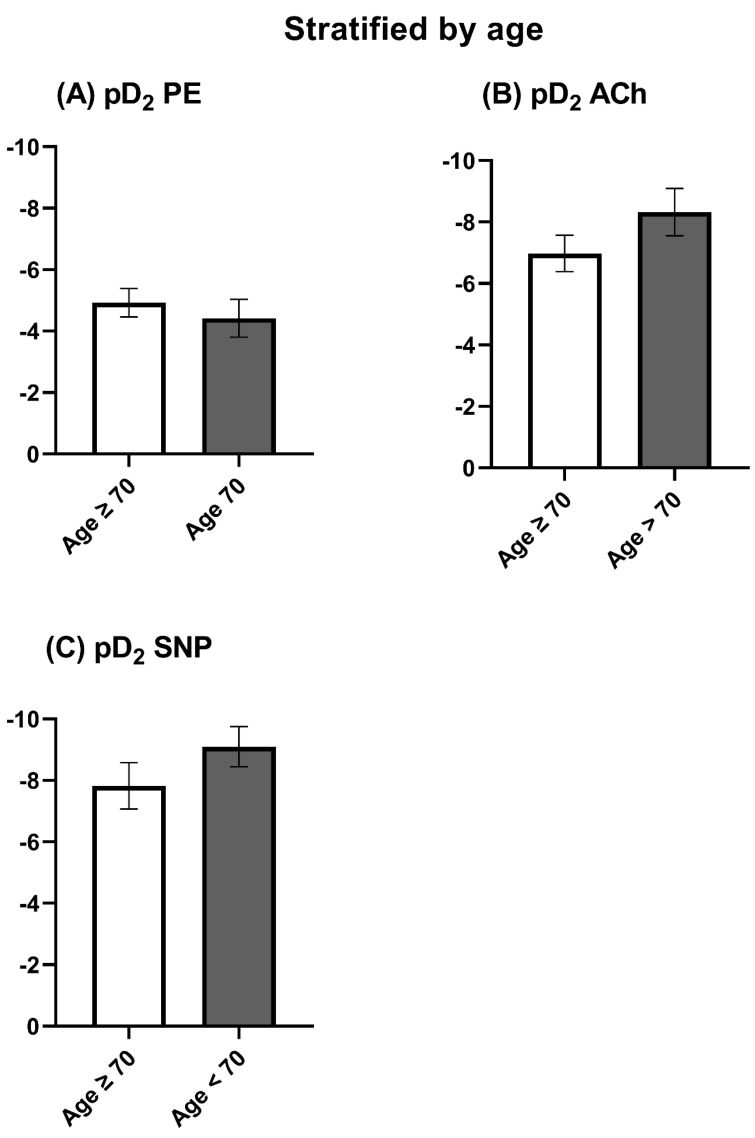
pD_2_ stratified by age. ACh: Acetylcholine. KCl: Potassium chloride. PE: Phenylephrine. SNP: Sodium nitroprusside. The data were compared using a two-tailed unpaired classical *t*-test in case of variance homogeneity and a Welch *t*-test in case of variance inhomogeneity.

The maximal contraction induced by KCl was tendentially stronger in patients ≥70 years of age (Figure 2A). However, the relative contraction induced by PE in % of KCl was similar between both groups (Figure 2B and Figure 3A). Nevertheless, patients <70 years of age showed a significantly higher endothelial-dependent relaxation compared to patients ≥ 70 years (Figure 2C and Figure 3B). The endothelial-independent relaxation induced by SNP was similar between both groups (Figure 3C). The pD_2_ did not differ significantly but tended to be smaller in patients < 70 years (Figure 4).

### 3.3. Stratification by Bodyweight

Considering that bodyweight was significantly different between both groups and has been shown to have a significant impact on outcomes in cardiovascular medicine [8], we also investigated the effect of BW on the vasomotor function of the same segments of the saphenous vein grafts.

### 3.4. Patient Data

We compared the same patient data for the bodyweight stratification to the age stratification (Table 2). Both groups were comparable regarding all parameters except sex (female, 2.7% vs. 19.2%), LVEF, and obesity.

### 3.5. Vasomotor Function Stratified by Bodyweight

**Figure 5:**
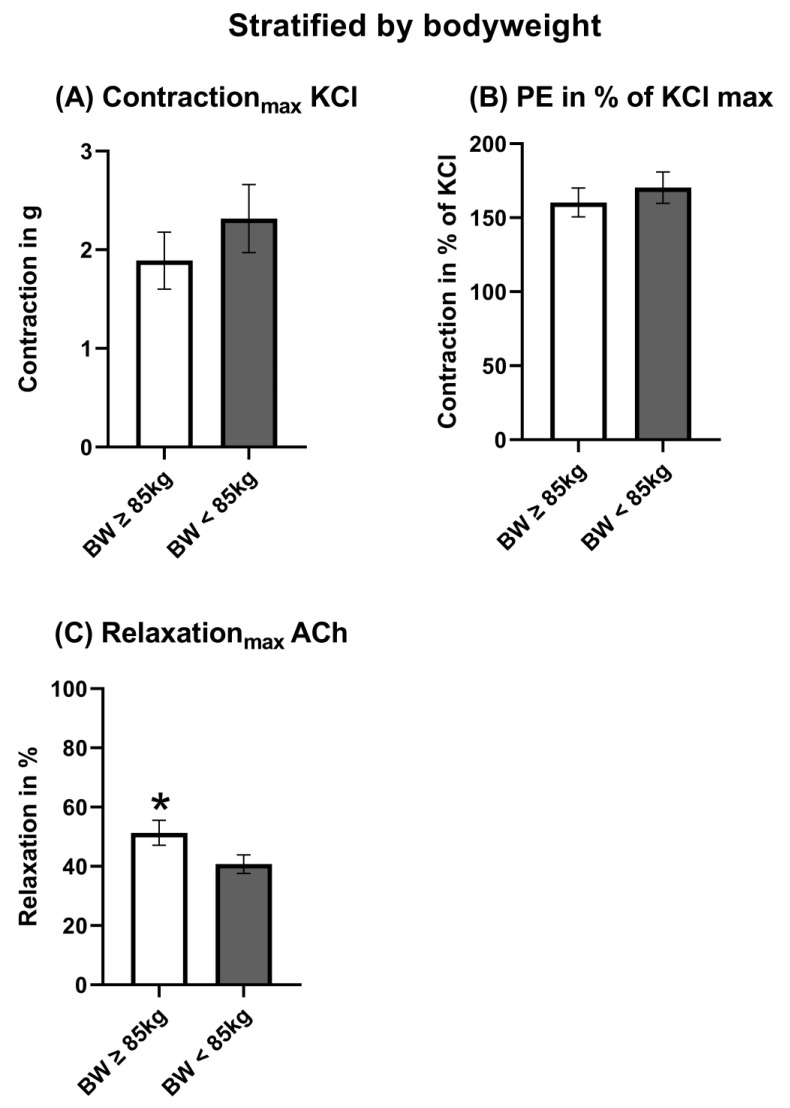
Maximal endothelial-dependent vasomotor response stratified by bodyweight. ACh: Acetylcholine. KCl: Potassium chloride. PE: Phenylephrine. The data were compared using a two-tailed unpaired classical *t*-test in case of variance homogeneity and a Welch *t*-test in case of variance inhomogeneity. * *p* < 0.05 compared to bodyweight <85 kg.

**Figure 6:**
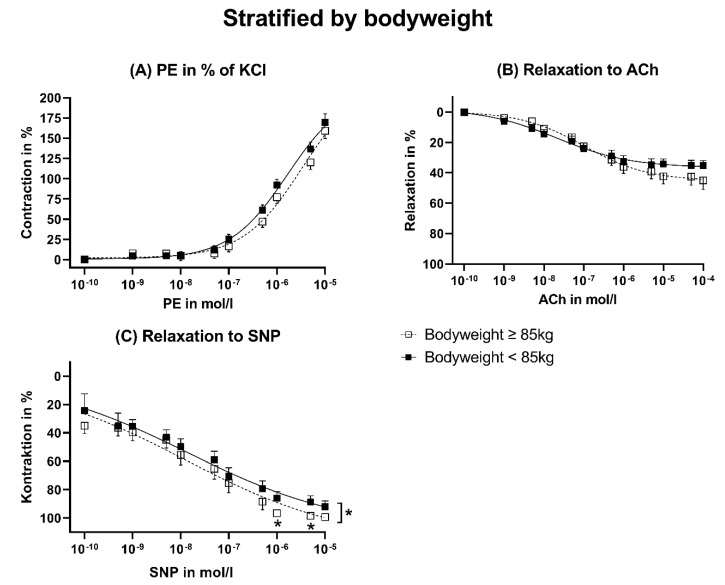
Dose-response curves stratified by bodyweight. ACh: Acetylcholine. KCl: Potassium chloride. PE: Phenylephrine. SNP: Sodium nitroprusside. The data were compared using a two-tailed unpaired classical *t*-test in case of variance homogeneity and a Welch *t*-test in case of variance inhomogeneity. * *p* < 0.05.

**Figure 7:**
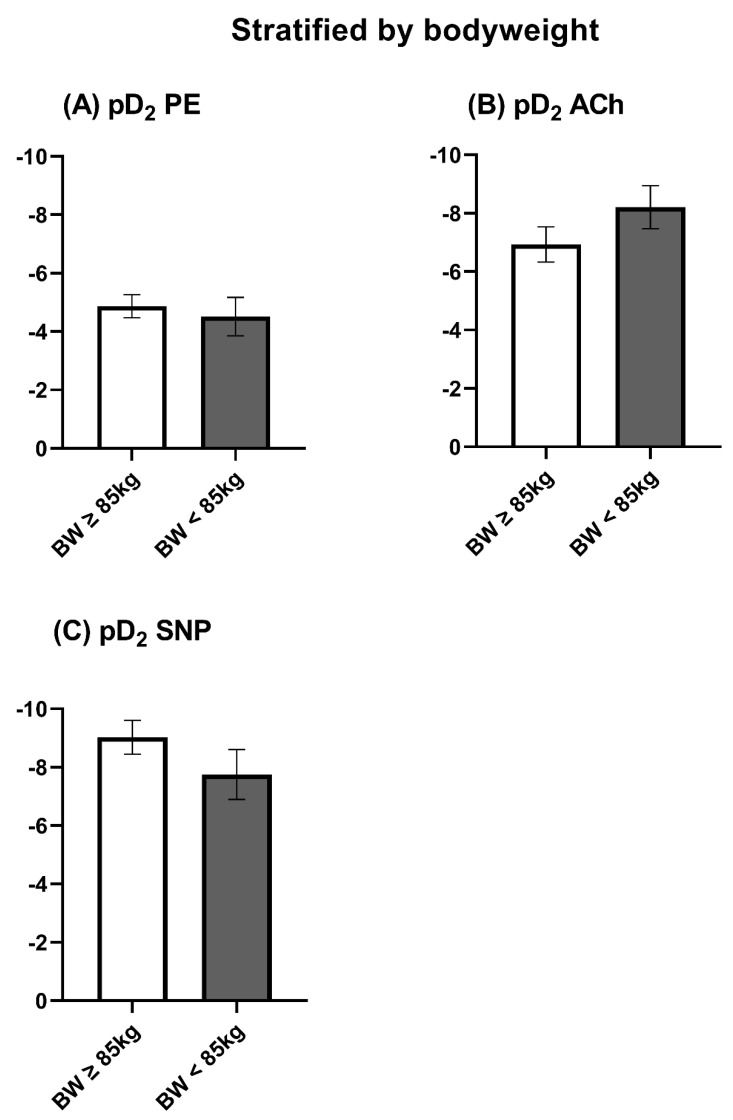
Maximal endothelial-dependent vasomotor response stratified by bodyweight. ACh: Acetylcholine. KCl: Potassium chloride. PE: Phenylephrine. The data were compared using a two-tailed unpaired classical *t*-test in case of variance homogeneity and a Welch *t*-test in case of variance inhomogeneity.

The maximal contraction induced by KCl was stronger, even when not significant, in the <85 kg of BW group (Figure 5A). However, the maximal and dose-dependent contractions induced by PE related to KCl were comparable between both groups (Figure 5B and Figure 6A). The maximal endothelial-dependent relaxation induced by ACh was significantly higher in patients ≥ 85 kg (Figure 5C). Nevertheless, the absolute differences were low (Figure 5C). The endothelial-independent relaxation induced by SNP was also not majorly, but significantly, higher in patients with a bodyweight ≥ 85 kg (Figure 6C). Accordingly, the pD_2_ was slightly lower in response to SNP (Figure 7C). The pD_2_ for PE and ACh did not show significant differences between both bodyweight groups (Figure 7A,B).

## 4. Discussion

### 4.1. Relevance of Initial Vascular Function of the Conduit

Myocardial malperfusion, early postoperative graft occlusion, and shortened long-term graft patency are dreaded complications after CABG surgery. The initial endothelial injury of the conduit contributes to all of these complications. It is known that different factors contribute to preserving an intact endothelial layer, such as storage solutions and harvesting techniques, e.g., the no-touch technique. However, many other potential patient-dependent and -independent contributors to conduit endothelial function presumably exist. Nevertheless, the final endothelial function of the harvested graft prior to anastomosation cannot be assessed intraoperatively. Consequently, respective data do not exist. Thus, we investigated the impact of two potential contributors, age and bodyweight, on the endothelial function or dysfunction of saphenous vein bypass grafts in patients with CAD treated with CABG.

### 4.2. Age

In patients ≥ 70 years of age, endothelial-dependent relaxation was significantly impaired. Both groups were comparable with regard to the included patient characteristics, such as sex, comorbidities, and medications.

The fact that the response to PE related to KCl is similar in both groups implies that the differing maximal response to KCl is independent of the endothelial integrity and is based on downstream effects. Our results suggest a comparable endothelial integrity between both age groups.

Reasons for vascular endothelial dysfunction can be diverse, especially considering age and comorbidities. Arterial vessels are characterized by wall thickening, mainly of the intima, increased vascular calcification, and stiffness with increasing age [9]. These effects might differ in veins but still need to be investigated, ideally in direct comparison to arterial grafts, such as internal mammary arteries.

Vascular calcification is a part of the endothelial phenotype switching of arteries in old age [10]. It has been shown that vascular stiffness increases with age in arterial grafts, due to a switch in the elastin-to-collagen ratio [11]. However, vein conduits also consist of elastin and collagen [12]. In addition, advanced glycation end products (AGEs), which accumulate with age, reduce vascular elasticity as well. AGEs can link with collagen fibers, resulting in structurally dysfunctional collagen [13]. In addition to the passive effects that lead to vascular dysfunction in old age, age-associated endothelial dysfunction also leads to decreased vasorelaxation, as confirmed by our results. In old age, endothelial dysfunction is caused by impaired nitric oxide (NO) signaling. The underlying reasons are AGE-associated quenching of NO [14] and upregulated arginase activity in old age. Thus, less L-Arginine, a precursor substance for NO, is available for NO production [15]. Additionally, more endothelial reactive oxygen species are produced in old age that might damage the endothelium [16]. Endothelial apoptosis is also higher in old age [9]. These known vascular effects of aging might have caused the impaired endothelial-dependent relaxation of the saphenous vein grafts in the group aged ≥ 70 years. However, molecular analytical work still needs to be performed to identify the exact pathomechanisms. The observed effects of old age could be even more pronounced when comparing groups with a bigger age difference than 76.5 ± 0.6 (≥70 years group) versus 62.4 ± 1.2 years (<70 years group).

In all age stages, protecting the endothelium in bypass grafts for CABG surgery is crucial to reestablishing post-stenotic myocardial perfusion and maintaining high graft blood flow long-term.

The literature shows that mainly three topics have been investigated regarding the protection of the endothelium of venous conduits in CABG: (1) pressure distension during vein graft preparation [2]; (2) mechanical injury during harvesting, comparing the no-touch technique to conventional harvesting [17,18]; and (3) storage solutions [19,20] to reduce oxidative stress [21], apoptosis [22], and wall thickening [23], improve eNOS expression, and affect NfκB and MAPK signaling [24,25]. One study also investigated the potential role of statin therapy [26]. Nevertheless, patients who require CABG surgery can be very diverse and might be characterized by multiple comorbidities and medications that potentially affect the bypass conduit.

CABG surgery is the gold standard therapy to treat CAD. Thus, a huge effort should be made to improve results after CABG and identify risk factors for conduit injury. Considering the increasing age of CABG patients [5] and the decreased endothelial function of saphenous vein grafts in old age, the aspect of aging should receive significant focus while improving graft protection. Vulnerable or partially dysfunctional venous conduits from old patients might need to be treated differently during storage. Graft protection might also include the modification of preservation solutions according to the patient’s age and comorbidities, such as by senotherapeutics [27].

### 4.3. Bodyweight

In many interventional clinical studies in cardiovascular medicine, the impact of bodyweight on the outcome has been investigated. These studies have shown that being overweight, to a certain degree, positively affects the outcome after CABG surgery [8]. A potential reason could have been a difference in graft quality based on bodyweight. Nevertheless, the impact of bodyweight on conduit endothelial function has not been investigated yet. Our results showed greater relaxation in patients ≥ 85 kg. Both groups were comparable regarding all assessed parameters except obesity, LVEF, and sex. Nevertheless, it is reasonable to assume that obesity and bodyweight are associated with each other. In addition, it is unlikely that LVEF has an impact on saphenous vein relaxation and contraction. Matching both groups based on sex would be very interesting. However, the patient numbers are too low to perform statistical adjustments or propensity score matching. Whether the minor difference in endothelial relaxation is a major driver of the better outcome after CABG in patients who are slightly overweight remains to be investigated in the near future.

## 5. Conclusions

The saphenous veins are the most frequently used grafts in CABG. However, maintaining both short- and long-term graft patency is highly important to ensure perfusion of the post-stenotic myocardial area. Multiple factors, such as comorbidities and age, might impact graft patency. Our results show that the endothelial function of saphenous vein grafts decreases in old age. Consequently, age is an essential factor that should be considered when developing new methods of graft protection.

## Figures and Tables

**Table 1:** Age. BMI: Body mass index. LVEF: Left-ventricular ejection fraction. N/A: Not available. NOAC: non vitamin K antagonist oral anticoagulants. NYHA: New York Heart Association. PAD: Peripheral artery disease. The data were compared using a χ^2^ test for categorical data or a two-tailed unpaired classical *t*-test in case of variance homogeneity and a Welch *t*-test in case of variance inhomogeneity.

Parameter	Category	Age ≥ 70 Years *n* = 40 (%)	Age < 70 Years *n* = 33 (%)	*p*-Value
Sex	female *(n = 16)*	9 (12.3)	7 (9.6)	0.9
	male *(n = 57)*	31 (42.5)	26 (35.6)	
Bodyweight in kg		79.9 ± 2.3	91.3 ± 3.0	0.003
	≥85 kg *(n = 37)*	17 (23.3)	20 (27.4)	0.12
	<85 kg *(n = 36)*	23 (31.5)	13 (17.8)	
BMI		27.5 ± 0.7	30.5 ± 0.7	0.89
*Graft characteristics*				
Storage solution	Tiprotec^®^ *(n = 21)*	13 (18.1)	8 (11.1)	0.34
	NaCl-heparin *(n = 16)*	8 (11.1)	8 (11.1)	
	Ringer-heparin *(n = 13)*	6 (8.3)	7 (9.7)	
	Blood-heparin *(n = 6)*	1 (1.4)	5 (6.9)	
	NaCl *(n = 11)*	8 (11.1)	3 (4.2)	
	Ringer *(n = 5)*	3 (4.2)	2 (2.8)	
Graft diameter in mm		3.4 ± 0.2	3.1 ± 0.2	0.3
	≥3 mm *(n = 28)*	18 (26.5)	20 (29.4)	0.97
	<3 mm *(n = 38)*	19 (27.9)	11 (16.1)	
*Comorbidities*				
Arterial hypertension	Existent *(n= 69)*	38 (52.1)	31 (42.5)	0.84
	Non-existent *(n = 4)*	2 (2.7)	2 (2.7)	
NYHA-classification	NYHA I *(n = 3)*	1 (1.4)	2 (2.7)	0.52
	NYHA II *(n = 13)*	6 (8.2)	7 (9.5)	
	NYHA III *(n = 18)*	8 (11.0)	10 (13.7)	
	NYHA IV *(n = 2)*	1 (1.4)	1 (1.4)	
	N/A *(n = 37)*	24 (32.9)	13 (17.8)	
LVEF in %	<30% *(n = 4)*	3 (4.1)	1 (1.4)	0.71
	30–50% *(n = 28)*	15 (20.5)	13 (17.8)	
	>50% *(n = 41)*	22 (30.1)	19 (26)	
PAD	Existent *(n = 6)*	2 (2.7)	4 (5.5)	0.27
	Non-existent (*n = 67*)	38 (52.0)	29 (39.7)	
Obesity	Class 1 *(n = 21)*	8 (11.0)	13 (17.8)	0.36
	Class 2 (*n= 6)*	3 (4.1)	3 (4.1)	
	Class 3 *(n = 1)*	0 (0)	1 (1.3)	
	Non-existent *(n = 34)*	20 (27.4)	14 (19.2)	
Hyperlipoproteinemia	Existent *(n = 32)*	19 (26.0)	13 (17.8)	0.49
	Non-existent *(n = 40)*	21 (28.8)	20 (27.4)	
Chronic kidney insufficiency	Stage 1 *(n = 5)*	5 (6.8)	1 (1.4)	0.48
	Stage 2 *(n = 5)*	3 (4.1)	2 (2.7)	
	Stage 3 *(n = 15)*	7 (9.6)	8 (11.0)	
	Non-existent *(n = 47)*	25 (34.2)	22 (30.1)	
Diabetes mellitus	Type 1 *(n = 1)*	1 (1.4)	0 (0)	0.48
	Type 2 *(n = 32)*	19 (26.0)	13 (17.8)	
	Non-existent *(n = 40)*	20 (27.4)	20 (27.4)	
*Medications*				
Oral antidiabetics	Administered *(n = 24)*	14 (19.2)	10 (13.7)	0.26
	Not administered *(n = 6)*	5 (6.8)	1 (1.4)	
	No diabetes *(n = 43)*	21 (28.8)	22 (30.1)	
Antiplatelet drugs	Administered *(n = 59)*	32 (43.8)	27 (37.0)	0.84
	Not administered *(n = 14)*	8 (11.0)	6 (8.2)	
NOACs	Administered *(n = 10)*	5 (6.8)	5 (6.8)	0.74
	Not administered *(n = 63)*	35 (47.9)	28 (38.4)	
Diuretics	Administered *(n = 47)*	27 (37.0)	20 (27.4)	0.54
	Not administered *(n = 26)*	13 (17.8)	13 (17.8)	
Statins	Administered *(n = 55)*	27 (37.0)	28 (38.4)	0.09
	Not administered *(n = 18)*	13 (17.8)	5 (6.8)	

**Table 2:** Bodyweight. BW: Bodyweight. BMI: Body mass index. LVEF: Left ventricular ejection fraction. N/A: Not available. NOAC: non vitamin K antagonist oral anticoagulants. NYHA: New York Heart Association. PAD: Peripheral artery disease. The data were compared using a χ^2^ test for categorical data or a two-tailed unpaired classical *t*-test in case of variance homogeneity and a Welch *t*-test in case of variance inhomogeneity.

Parameter	Category	BW ≥ 85 kg *n* = 37 (%)	BW < 85 kg *n* = 36 (%)	*p*-Wert
Sex	Female *(n = 16)*	2 (2.7)	14 (19.2)	0.001
	Male *(n = 57)*	35 (47.9)	22 (30.1)	
Bodyweight in kg		69.3 ± 1.6	70.9 ± 1.4	0.43
	≥70 years (*n = 40)*	17 (23.3)	23 (31.5)	0.12
	<70 years *(n = 33)*	20 (27.4)	13 (17.8)	
BMI		31.3 ± 0.6	26.1 ± 0.7	0.16
*Graft characteristics*				
Storage solution	Tiprotec^®^ (*n = 21)*	11 (15.3)	10 (13.9)	0.57
	NaCl-heparin *(n = 16)*	10 (13.9)	6 (8.3)	
	Ringer-heparin *(n = 13)*	4 (5.6)	9 (12.5)	
	Blood-heparin *(n = 6)*	4 (5.6)	2 (2.8)	
	NaCl *(n = 11)*	6 (8.3)	5 (6.9)	
	Ringer *(n = 5)*	2 (2.8)	3 (4.2)	
Graft diameter in mm		3.2 ± 0.2	3.3 ± 0.2	0.93
	≥3 mm *(n = 28)*	18 (27.3)	20 (30.3)	0.62
	<3 mm *(n = 38)*	15 (22.7)	13 (19.7)	
*Comorbidities*				
Arterial hypertension	Existent *(n = 69)*	34 (46.6)	35 (47.9)	0.32
	Non-existent *(n = 4)*	3 (4.1)	1 (1.3)	
NYHA-classification	NYHA I *(n = 3)*	1 (1.3)	2 (2.7)	0.55
	NYHA II *(n = 13)*	7 (9.5)	6 (8.2)	
	NYHA III (*n = 18)*	12 (16.4)	6 (8.2)	
	NYHA IV *(n = 2)*	1 (1.3)	1 (1.3)	
	N/A *(n = 37)*	16 (21.9)	21 (28.8)	
LVEF in %	<30% *(n = 4)*	1 (1.4)	3 (4.1)	0.004
	30–50% *(n = 28)*	21 (28.8)	7 (9.6)	
	>50% *(n = 41)*	15 (20.5)	26 (35.6)	
PAD	Existent *(n = 6)*	2 (2.7)	4 (5.4)	0.38
	Non-existent *(n = 67)*	35 (47.9)	32 (43.8)	
Obesity	Class 1 *(n = 21)*	17 (23.3)	4 (5.5)	0.001
	Class 2 *(n = 6)*	5 (6.8)	1 (1.3)	
	Class 3 *(n = 1)*	1 (1.3)	0 (0)	
	Non-existent *(n = 45)*	14 (19.2)	31 (42.5)	
Hyperlipoproteinemia	Existent *(n = 32)*	15 (20.5)	17 (23.3)	0.57
	Non-existent *(n = 41)*	22 (30.1)	19 (26.0)	
Chronic kidney insufficiency	Stage 1 *(n = 6)*	1 (1.4)	5 (6.8)	0.30
	Stage 2 *(n = 5)*	2 (2.7)	3 (4.1)	
	Stage 3 *(n = 15)*	9 (12.3)	6 (8.2)	
	Non-existent *(n = 47)*	25 (34.2)	22 (30.1)	
Diabetes mellitus	Type 1 *(n = 1)*	0 (0)	1 (1.4)	0.47
	Type 2 *(n = 32)*	15 (20.5)	17 (23.3)	
	Non-existent *(n = 40)*	22 (30.1)	18 (24.7)	
*Medications*				
Oral antidiabetics	Administered *(n = 24)*	12 (16.4)	12 (16.4)	0.99
	Not administered *(n = 6)*	3 (4.1)	3 (4.1)	
	No diabetes *(n = 43)*	22 (30.1)	21 (28.8)	
Antiplatelet drugs	Administered *(n = 59)*	29 (39.7)	30 (41.1)	0.59
	Not administered *(n = 14)*	8 (11.0)	6 (8.2)	
NOACs	Administered *(n = 10)*	5 (6.8)	5 (6.8)	0.96
	Not administered *(n = 63)*	32 (43.8)	31 (42.5)	
Diuretics	Administered *(n = 47)*	24 (32.9)	23 (31.5)	0.93
	Not administered *(n = 26)*	13 (17.8)	13 (17.8)	
Statins	Administered *(n = 55)*	30 (41.1)	25 (34.2)	0.25
	Not administered *(n = 18)*	7 (9.6)	11 (15.1)	

## Data Availability

Available on request.

## Abbreviations

ACh	Acetylcholine
AGE	Advanced glycation endproducts
CABG	Coronary artery bypass grafting
CAD	Coronary artery disease
KCl	Potassium chloride
KHS	Krebs–Henseleit solution
NO	Nitric oxide
PE	Phenylephrine
SNP	Sodium nitroprusside
